# Early identification of individuals at risk for loss to follow-up of tuberculosis treatment: A generalised hierarchical analysis

**DOI:** 10.1016/j.heliyon.2021.e06788

**Published:** 2021-04-20

**Authors:** Shirley Verônica Melo Almeida Lima, Karina Conceição Gomes Machado de Araújo, Marco Antonio Prado Nunes, Carla Nunes

**Affiliations:** a Post-Graduate Program in Health Sciences, Federal University of Sergipe, Brazil; b NOVA National School of Public Health, Universidade NOVA de Lisboa, Portugal; c Public Health Research Centre, Universidade NOVA de Lisboa, Portugal

**Keywords:** Tuberculosis, Therapy, Risk factors, Social determinants of health, Epidemiology

## Abstract

**Background:**

We characterise the loss to follow-up (locally termed abandoned) of tuberculosis treatment with individual and ecological health determinants and to identify the predictive capacity of these risk factors.

**Methods:**

A cohort study with individual and ecological characterisation of patients diagnosed with tuberculosis in Sergipe/Brazil from 2015 to 2018 with either loss to follow-up or completion of treatment as a therapeutic outcome was performed. The examined variables were based on the social determinants of health with descriptive analysis, binary logistic regression, a generalised hierarchical model and graphical presentation using a nomogram.

**Results:**

The loss to follow-up accounted for 18.21% of the 2,449 studied cases. The characteristics revealed that the highest abandonment percentages were people who: were male (20.0%), had black skin colour (20.3%), were aged 20–39 years (21.8%), had 4–7 years of schooling (23.6%), re-entered treatment after abandonment (36.5%), used alcohol (31.0%), used drugs (39.3%), were smokers (26.5%) and were homeless (55.4%). The ecological characteristics showed that individuals living in municipalities with a high human development index (HDI; odds ratio [OR]: 1.91) and high-income inequality (OR: 1.81) had a greater chance of not finishing the treatment. Most of these variables were identified as predictors in the generalised hierarchical model; the receiver operating characteristic curve (ROC) curve had 0.771 precision and 84.0% accuracy.

**Conclusion:**

The group of identified characteristics influenced the loss to follow-up of tuberculosis treatment. This data provides evidence for the early identification of individuals who are at greater risk of abandoning tuberculosis treatment.

## 1 Introduction

Tuberculosis (TB) has a worldwide impact on public health, since it is the infectious disease that kills the most people globally [1] and is an epidemic still neglected [2] and not solved in the 21st century. The severity of the epidemic is highly variable between countries, and the World Health Organization (WHO) reinforces the implementation of strategies and actions towards the end of the epidemic by 2030, considering the pillars of early diagnosis, treatment and completion of treatment [1].

TB treatment lasts for at least 6 months, so it is essential that the professional guides the patient through the health service from diagnosis to completion of treatment. Worldwide, out of the 30 priority countries for TB control actions, only nine achieved a 90% treatment success rate. Countries such as Angola, Brazil, the Central African Republic, Liberia and Papua New Guinea showed greater than 10% loss following treatment [1, 3]. In general, treatment success has increased, but loss to follow-up remains high in the Americas (26%). Brazil has abandonment rates from 38% [4] to 42% [5], which are considered high compared to what is proposed by the WHO (<5%; 1,6).

The loss to follow-up in treatment can increase the transmissibility of the disease. Therefore, TB completion of treatment is extremely important for both the patient and the community in which s/he lives [6]. The loss to follow-up is considered when an individual who after starting treatment stops going to the health service for more than thirty consecutive days after the date scheduled for her/his return [3]. Understanding the health-disease process, especially in TB, requires knowledge that reflects the connection of the social context with the biological aspect of the disease and its transmissibility [7]. The loss to follow-up in treatment is considered one of the main obstacles and challenges in the fight against TB, and it has direct consequences on fast transmission of the disease, elevated treatment cost, mortality and increased recurrence rates [8]. The analysed region in this study (Sergipe) has the highest percentage of abandonment in the Brazilian Northeast [9].

The failures in global TB control have contributed to increased loss to follow-up, mortality and drug resistance [10]. TB treatment loss to follow-up is still prevalent on the world stage and is a serious problem for global public health [11]. Thus, the Sustainable Development Goals (SDG) ratify the need for improvements in the coverage of health services and strategies that include socioeconomic determinants [1, 12].

When we consider the TB therapeutic process, we understand that disease morbidity and mortality can be a reflection of the living conditions of the population associated with individual factors and health service quality. The reflection of these dynamics is important to establish and recognise relationships between risk factors and the TB therapeutic outcome. Therefore, the interaction of these characteristics can be understood in a broader sense and not only by individual-level factors.

The completion of treatment for TB requires specific monitoring by health services in order to avoid therapeutic failures. In this sense, it is crucial to understand the main factors that can influence treatment loss to follow-up. Thus, this study aimed to characterise the loss to follow-up of TB treatment using individual and ecological health determinants and to identify the predictive capacity of these risk factors.

## 2 Methods

### 2.1 Study design

This cohort study utilised ecological and individual characterisation of patients diagnosed with TB in Sergipe, Brazil from 2015 to 2018 whose therapeutic outcome was completion of treatment or loss to follow-up. The other therapeutic outcomes, such as deaths (n = 51), transfers (n = 48), diagnostic change (n = 23), cases still in treatment (n = 17) and drug-resistant TB (n = 6), were excluded from the analysis. Sergipe is one of the 27 units of the Federative Republic of Brazil; it is the smallest of the Brazilian states, occupying a total area of 21.910 km^2^. The population in 2018 was estimated at 2,278,308 inhabitants, with a population density of 94.3 inhabitants/km^2^. It has 75 municipalities, and Aracaju is the state capital [13]. It is situated in the Northeast Region and is bordered to the north by the state of Alagoas and the São Francisco River. To the south and west, it is bordered by the state of Bahia and to the east by the Atlantic Ocean [14].

### 2.2 Data source and definition of variables

The research used two data sources. Individual data were extracted from the Information System of Notification Diseases (SINAN) provided by the State Health Department of Sergipe. The ecological data were supported by the Brazilian Institute of Geography and Statistics (IBGE), referring to the 2010 census.

Individual characterisation of the cases diagnosed with TB was explored by variables contained in SINAN, namely: sex (male or female); skin colour (white, black, Asian, brown, indigenous or ignored); age group (0–19, 20–39, 40–59 or 60 years and over); education in years (unknown, 1–3, 4–7, 8–11 or 12 and more); area of residence (urban or rural); case type, considering the existence of previous treatment (new case, relapse, re-entry after loss to follow-up or transfer); form of TB (pulmonary or extrapulmonary); AIDS (yes or no); alcohol use (yes or no); diabetes (yes or no); mental disorder (yes or no); drug use (yes or no); smoking (yes or no); deprived of liberty (yes or no); homeless (yes or no); immigrant (yes or no); and recipient of government income (yes or no).

Considering the specific TB context, for ecological and contextual characterisation two social indexes that represent world parameters were used: the human development index (HDI), denoted as very high, high, medium or low levels composed of the dimensions of education, longevity and income of individuals [15], and the Gini index as a measure of income inequality between 0 (zero), representing complete equality, and 1 (one), which represents complete inequality with categories defined in low, moderate and high inequality [16].

**Figure 1:**
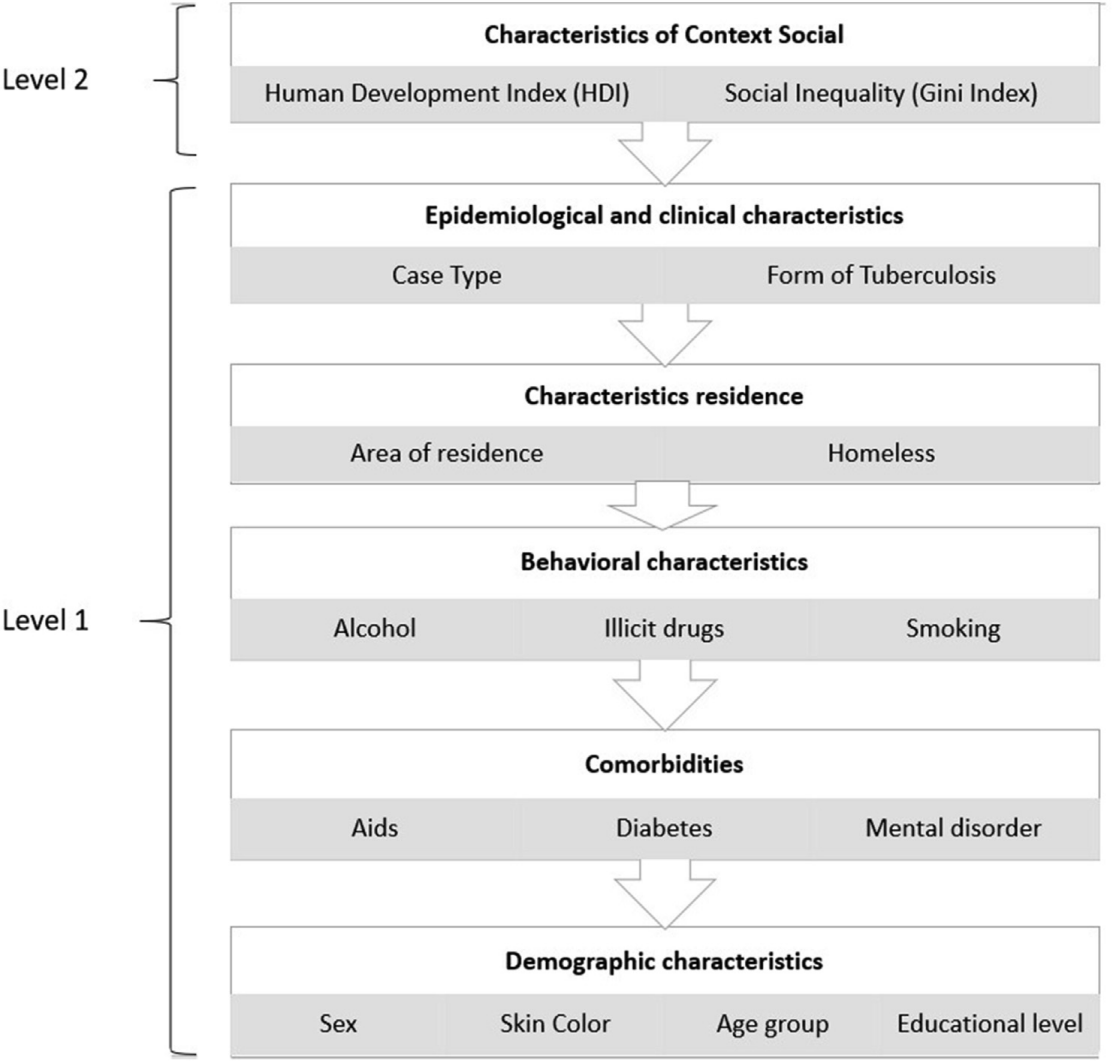
Hierarchical structure considering individuals and groups aligned at different levels.

Hierarchical definition of the variables was based on the theoretical model proposed by Dahlgren and Whitehead [17] and Solar and Irwin [18]. The criteria of choice were based on the social determinants of health (SDH) that show different levels of comprehensiveness, starting with individual determinants (in a more proximal layer) to collective determinants of society as a more distal layer (Figure 1). These conditions highlight the possible influence of social determinants on health conditions [19], specifically TB for this study.

The dependent dichotomic variable was the outcome of the case diagnosed with TB: completion of treatment or loss to follow-up.

### 2.3 Data analysis and ethical aspects

The statistical treatment of data reflects two approaches, namely individual and ecological. The data was initially stored in Microsoft Excel (Windows version 2013, Microsoft Corporation; Redmond, WA, USA). Analyses were performed using Statistical Package for the Social Sciences version 24.0 (SPSS Inc., Chicago, IL, USA) or R version 3.6.0 (Copyright 2019, The R Foundation for Statistical Computing General Public License).

Initially, individual and ecological descriptive analyses were shown in absolute numbers and simple frequencies considering TB case outcome (completion of treatment or loss to follow-up). We performed a bivariate logistic regression for each individual and context variable in order to determine the Beta (B) odds ratio (OR) and its respective 95% confidence interval (CI), which means the estimated regression coefficient and the respective statistics, derived from the logistic regression model that predicts the dependent variable from the constant and the independent variables. The logistic regression equation allowed estimating the probability of each individual belonging to a certain group (loss to follow-up or completion of treatment).

To construct the hierarchical multiple model, the individual and ecological variables that showed statistical significance with an error probability less than 5% in the bivariate logistic regression were considered. The generalised hierarchical model was applied [20], and the codes for each municipality with reference to each TB case were used as the basic structure for the model. The fixed effects were represented by the independent variables at their individual and ecological levels, and the random effect was at the municipal level.

We used the backward method considering the predictive capacity of the significant variables and utilised the receiver operating characteristic “*Receiver Operating Characteristic*” (ROC) curve to measure the sensitivity and accuracy of the model. This method identified the determinant factors for TB treatment loss to follow-up with the construction of a risk model. The choice of the best statistical performance was selected, the logistic model that presented the best mathematical behavior, considering the performance and the area of the curve ROC in multi level model.

The graphical representation of the predictive model is presented using a nomogram that favours early identification of individuals at risk for treatment loss to follow-up and provides good conditions for use in health services. The nomogram contains a set of scales: one for each variable identified in the predictive model, two for the calculation of the score and one end that allows visualising the probability of abandonment [6, 21, 22, 23]. The output of scale third, should be interpreted in multiplication the number 0.1 to 0.9 for percentage risk of loss to follow-up from 10% to 90%.

The nomogram is a facilitating tool for use of health professionals in order to analyze the risk that the patient may have for loss of follow-up in the treatment of tuberculosis. The nomogram is indicating the probability of loss to follow-up in association with the person's risk factors. This risk is analyzed based on the risk factors of each individual and the sum of these factors may result in a greater or lesser risk of loss to follow-up.

Health professionals should use the nomogram as follows: in a hypothetical situation the patient has been diagnosed with TB and the nomogram will be applied to check the likelihood of loss to TB follow-up.

Example: Individual characteristics (first scale): black skin colour = 20 points; 30–39 years old = 40 points; 1 to 3 education years = 100 points; AIDS (No) = 0 points; Alcohol use (Yes) = 20 points; Drugs use (No) = 0 points; residence in urban area = 20 points; Homeless (No) = 0 points; Re-entry after abandonment = 87 points; Resides in an area with a high HDI = 20 points; Resides in an area with a high Gini Index = 0 points. Total (second scale): 307 points. To interpret the nomogram (third scale), this hypothetical patient has between 0.6 and 0.7 chance of abandonment, that is, a 60%–70% probability of losing TB follow-up.

Regarding ethical aspects, the rules described by Resolution 510/2016 of the National Health Council were respected, and the research was approved by the Ethics and Research Committee on Human (No. 1,768,049). The rules of the Helsinki Convention were followed.

## 3 Results

The descriptive analysis was supported by 2,449 TB occurrences with therapeutic outcome of loss to follow-up or completion of treatment. However, 51 people who died, 48 transfers to another state, 23 diagnostic change, 17 cases still in treatment and 6 people with drug resistant TB were excluded from the analysis. The loss to follow-up represented 18.21% of the analysed cases. Table 1 shows that loss to follow-up was prevalent among males (20.03%), individuals with black (20.34%) and brown (19.55%) skin colour, those aged 20 39 years (21.87%) and persons with none or only a few years of education. Individuals who resided in urban areas (19.64%), those who restarted treatment after loss to follow-up (58.13%) and those diagnosed with pulmonary TB (19.08%) had high abandonment rates. People with AIDS (42.33%), a mental disorder (36.82%) and behavioural characteristics such as alcohol use (31.05%) drug use (39.39%) or smoking (26.54%) were identified with higher rates of neglect (Table 1).

**Table 1:** Descriptive analysis of sociodemographic, epidemiological and social context characteristics among those diagnosed with TB, considering an outcome of completion of treatment or loss to follow-up, between 2015 and 2018 in Sergipe, Brazil.

Individual Characteristics		Completion of treatment/%	Loss to follow-up/%
Sex 2449∗ (0.0%)∗∗	Male	1399/80.02	350/20.03
	Female	604/86.38	96/13.74
Skin color 2352∗ (4.0%)∗∗	White	327/88.65	42/11.45
	Black	236/79.71	60/20.34
	Asian	23/82.16	5/17.91
	Brown	1332/80.56	322/19.55
	Indigenous	3/60.02	2/40.06
Age group (years) 2449∗ (0.0%)∗∗	0–19	164/86.36	26/13.72
	20–39	1020/78.25	284/21.87
	40–59	548/82.57	116/17.56
	60 or more	271/93.13	20/6.93
Education in years 2019∗ (17.6%)∗∗	Unknown	122/83.61	24/16.43
	1–3	494/78.91	132/21.12
	4–7	557/76.40	172/23.67
	8–11	367/88.02	50/12.04
	12 or more	98/97.06	3/3.06
Residence 2346∗ (4.2%)∗∗	Urban	1548/80.41	377/19.64
	Rural	374/88.89	47/11.27
Case type 2449∗ (0.1%)∗∗	New	1734/85.73	289/14.36
	Relapse	123/81.57	28/18.51
	Re-entry after loss to follow-up	78/41.91	108/58.13
	Transfer	66/78.69	18/21.42
Form of tuberculosis 2449∗ (0.0%)∗∗	Pulmonary	1761/81.06	414/19.08
	Extrapulmonary	242/88.33	32/11.77
AIDS 2146∗ (12.4%)∗∗	Yes	64/57.71	47/42.33
	No	1710/84.02	325/16.05
Alcohol 2417∗ (1.3%)∗∗	Yes	391/69.05	176/31.05
	No	1588/85.84	262/14.25
Diabetes 2422∗ (1.1%)∗∗	Yes	164/89.68	19/10.46
	No	1816/81.14	423/18.94
Mental disorder 2420∗ (1.2%)∗∗	Yes	36/63.22	21/36.82
	No	1942/82.26	421/17.87
Drugs use 2410∗ (1.6%)∗∗	Yes	230/60.78	149/39.39
	No	1744/85.91	287/14.12
Smoking 2422∗ (1.1%)∗∗	Yes	405/73.55	146/26.54
	No	1578/84.36	293/15.76
Population deprived of liberty 2436∗ (0.5%)∗∗	Yes	227/81.17	53/18.97
	No	1767/82.08	389/18.04
Homeless 2432∗ (0.7%)∗∗	Yes	33/44.61	41/55.44
	No	1957/83.05	401/17.08
Immigrants 2435∗ (0.6%)∗∗	Yes	9/90.06	1/10.02
	No	1982/81.71	443/18.35
Receives government benefit 2346∗ (4.2%)∗∗	Yes	165/86.82	25/13.23
	No	1758/81.56	398/18.53
**Characteristics of Context Social**			
Human development index 2449∗ (0.0%)∗∗	High	640/75.25	211/24.87
	Middle	1363/85.34	235/14.74
Gini index 2449∗ (0.0%)∗∗	High inequality	669/75.71	215/24.36
	Middle inequality	55/91.77	5/8.32
	Low inequality	1279/85.03	226/15.04

∗n/∗∗ Percentage of missing value.

Bivariate logistic regression revealed that the individuals who were male, had brown skin colour, were aged 20–39 years old, had no or only a few years of schooling, were urban dwellers or homeless, were diagnosed with pulmonary TB, re-entered after treatment drop out, had AIDS or mental disorder, used alcohol or illicit drugs and/or smoked were more likely to abandon treatment (Table 2).

**Table 2:** Bivariate logistic analysis of individual variables and social context among those diagnosed with TB who abandoned treatment between 2015 and 2018 in Sergipe, Brazil.

Independent Variables		B^a^	OR^b^	p-value	CI^c^ 95%
Sex 2449∗ (0.0%)∗∗	Male	0.454	1.574	<0.001	1.232–2.011
	Female(ref)				
Skin color 2352∗ (4.0%)∗∗	Black	0.683	1.979	0.002	1.290–3.038
	Asian	0.526	1.693	0.311	0.611–4.689
	Brown	0.632	1.882	<0.001	1.335–2.654
	Indigenous	1.647	5.190	0.076	0.843–31.965
	White(ref)			0.004	
Age group 2449∗ (0.0%)∗∗	0–19	0.765	2.148	0.015	1.162–3.971
	20–39	1.328	3.773	<0.001	2.351–6.053
	40–59	1.054	2.868	<0.001	1.746–4.712
	60 or more(ref)			<0.001	
Education in years 2019∗ (17.6%)∗∗	Unknown	1.860	6.426	0.003	1.880–21.971
	1–3	2.167	8.729	<0.001	2.723–27.976
	4–7	2.311	10.087	<0.001	3.158–32.224
	'8 a 11	1.493	4.450	0.014	1.359–14.573
	12 or more(ref)			<0.001	
Residence 2346∗ (4.2%)∗∗	Urban	0.662	1.938	<0.001	1.402–2.678
	Rural(ref)				
Case type 2449∗ (0.1%)∗∗	Relapse	0.312	1.366	0.154	0.890–2.097
	Re-entry after loss to follow-up	2.117	8.308	<0.001	6.052–11.404
	Transfer	0.492	1.636	0.072	0.958–2.796
	New(ref)			<0.001	
Form of tuberculosis 2449∗ (0.0%)∗∗	Pulmonary	0.575	1.778	0.003	1.211–2.610
	Extrapulmonary(ref)				
AIDS 2146∗ (12.4%)∗∗	Yes	1.352	3.864	<0.001	2.604–5.734
	No(ref)				
Alcohol 2417∗ (1.3%)∗∗	Yes	1.004	2.728	<0.001	2.188–3.402
	No(ref)				
Diabetes 2422∗ (1.1%)∗∗	Yes	-0.698	0.497	0.005	0.306–0.809
	No (ref)				
Mental disorder 2420∗ (1.2%)∗∗	Yes	0.990	2.691	<0.001	1.555–4.656
	No(ref)				
Drugs use 2410∗ (1.6%)∗∗	Yes	1.370	3.937	<0.001	3.094–5.009
	No(ref)				
Smoking 2422∗ (1.1%)∗∗	Yes	0.663	1.941	<0.001	1.548–2.435
	No(ref)				
Population deprived of liberty 2436∗ (0.5%)∗∗	Yes	0.059	1.061	0.717	0.771–1.458
	No(ref)				
Homeless 2432∗ (0.7%)∗∗	Yes	1.802	6.063	<0.001	3.787–9.709
	No(ref)				
Immigrants 2435∗ (0.6%)∗∗	Yes	-0.699	0.497	0.508	0.063–3.934
	No(ref)				
Receives government benefit 2346∗ (4.2%)∗∗	Yes(ref)				
	No	0.402	1.494	0.070	0.968–2.307
Human development index 2449∗(0.0%)∗∗	High	0.648	1.912	<0.001	1.553–2.355
	Middle(ref)				
Gini index 2449∗ (0.0%)∗∗	High inequality	0.598	1.819	<0.001	1.476–2.241
	Middle inequality	-0.665	0.514	0.160	0.204–1.299
	Low inequality(ref)			<0.001	

∗n/∗∗ Percentage of missing value/^a^ beta/^b^ Odds ratio/^c^ Confidence Interval/(ref): reference.

When considering the ecological characteristics, people who lived in municipalities with a high HDI were 1.91 times more likely to give up treatment when compared to those living in municipalities with a lower HDI. Regarding the Gini index, people living in municipalities with higher income inequality were 1.81 times more likely to abandon TB treatment than those who lived in municipalities with lower income inequality (Table 2).

Table 3 demonstrates the model with the best performance that identified 11 statistically significant variables predictive of TB treatment loss to follow-up. Predictive factors of the model included sociodemographic characteristics (skin colour, age group and schooling), existing comorbidity (AIDS), behavioural characteristics (alcohol and drug use), epidemiology (case type), living conditions (area of residence or homeless) and social context (HDI and Gini). For the parameters and performance of the hierarchical model, the ROC precision area was 0.771 (p < 0.001; 95% CI: 0.741–0.802) and the accuracy was 84.0%.

**Table 3:** Generalised hierarchical linear model with individual and ecological variables among those diagnosed with TB, considering a therapeutic outcome of completion of treatment or loss to follow-up, in Sergipe, Brazil.

Fixed Effects	ORa^a^	CI^b^ 95%	p- value	Coefficient
Intercept	0.004	(0.001–0.013)	<0.001	-5.524
**Level 1 (Individual Characteristics)**				
**Skin Color**				
Black	1.522	(1.016–2.282)	0.042	0.420
Asian	1.023	(0.411–2.549)	0.960	0.023
Brown	1.386	(1.032–1.862)	0.030	0.327
Indigenous	3.031	(0.571–16.094)	0.193	1.109
White(ref)				
**Age group (years)**				
0–19	2.029	(1.110–3.709)	0.022	0.707
20–39	2.340	(1.398–3.916)	<0.001	0.850
40–59	1.482	(0.835–2.629)	0.179	0.393
60 or more(ref)				
**Education in years**				
Unknown	7.780	(2.666–22.704)	<0.001	2.052
1–3	7.610	(2.764–20.957)	<0.001	2.030
4–7	8.116	(3.539–18.611)	<0.001	2.094
8 a 11	5.055	(2.328–10.976)	<0.001	1.620
12 or more(ref)				
**AIDS**				
Yes	2.617	(1.309–5.232)	0.007	0.962
No(ref)				
**Alcohol**				
Yes	1.608	(1.001–2.584)	0.049	0.475
No(ref)				
**Drugs use**				
Yes	1.645	(1.145–2.363)	0.007	0.498
No(ref)				
**Residence**				
Urban	1.583	(1.092–2.297)	0.015	0.460
Rural(ref)				
**Homeless**				
Yes	2.443	(1.464–4.074)	<0.001	0.893
No(ref)				
**Case type**				
Relapse	0.949	(0.535–1.683)	0.857	-0.052
Re-entry after loss to follow-up	5.751	(4.322–7.652)	<0.001	1.749
Transfer	2.310	(1.145–4.659)	0.019	0.837
New(ref)				
**Level 2 (Characteristics of Context Social)**				
**Human development index**				
High	1.591	(1.345–1.881)	<0.001	0.464
Middle(ref)				
**Gini index**				
High inequality	0.792	(0.619–1.013)	0.063	-0.233
Middle inequality	0.860	(0.378–1.954)	0.718	-0.151
Low inequality(ref)				

^a^ Odds Ratio Adjusted/^b^ Confidence Interval/(ref): reference.

**Figure 2:**
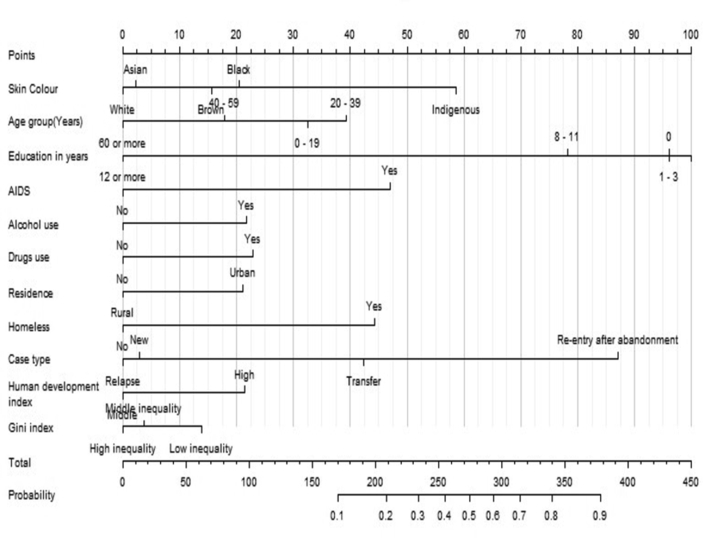
Nomogram with risk factors for tuberculosis treatment loss to follow-up in Brazil.

The nomogram was constructed based on the predictive variables of the generalised hierarchical model (Figure 2). The graph objectively shows the risk variables for TB treatment loss to follow-up.

## 4 Discussion

In our sample, TB treatment loss to follow-up was 18.21%, a value much higher than the WHO's proposal, which recommends abandonment of less than 5% [3, 24]. The predictive model identified that demographic, behavioural and epidemiological characteristics, existing comorbidities and the social context influence treatment loss to follow-up. The constructed nomogram allows the identification of patients at increased risk for loss to follow-up. In the multivariate context, individuals with black (OR: 1.52) or brown skin (OR: 1.38), aged 20–39 years (OR: 2.34), without schooling (OR: 7.78) or with 4–7 years of school (OR: 8.11) showed significant probabilities of not completing treatment. These findings are in accordance with the literature [25, 26, 27].

Individuals who acquire TB and already had AIDS as a comorbidity were 2.61 times more likely to abandon TB treatment compared to those who did not. Comorbidities may directly influence the TB therapeutic outcome, and people with immunosuppressive diseases are historically more likely to become ill with TB, especially those infected with HIV [3]. In this context, the individuality of these subjects should be treated in a special way by health professionals in order to minimise cases of loss to follow-up.

In addition to comorbidities, individual behaviours and lifestyle may compromise treatment success. Our study corroborates the literature [6] that identifies the use of alcohol and illicit drugs indicates a greater probability of loss to follow-up compared to those who do not use them. A study developed in Russia ratifies the importance of this group when it identified that interventions to reduce alcohol consumption associated with nutritional support and involving the patient with her/his disease improves adherence to TB treatment [28].

Homeless patients were 2.44 times more likely to abandon TB treatment when compared to those living in a household. A previous study identified that homeless individuals are 56 times more likely to become ill with TB [3]. The literature states that the health conditions of populations are directly related to the context in which they live and the position of individuals in the social pyramid [29]. Homeless patients are not a new phenomenon and this reality demands changes [30]. Therefore, the impact of social determinants on maintaining TB transmission is crucially important [31].

The social characteristics about TB treatment loss to follow-up are highlighted worldwide [11] and they are not different from the Brazilian reality. Indeed, we identified that individuals living in a municipality with a high HDI were 1.91 times more likely to not finish TB treatment when compared to municipalities with a lower HDI. This fact can be explained by the accelerated urbanisation of large cities. Further, although cities have high levels of development in education, longevity and income, there is a large discrepancy between living conditions. The association of the high HDI with loss to follow-up also can be justified because cities with a high human development index also contain large pockets of poverty and persistent peripheral areas. Thus, these situations of high percentages in the loss to follow-up can be blindfolded and covered up by major economic development within cities and states.

Income inequality, expressed by the Gini index, explains this reality when it revealed that people living in municipalities with high income inequality were 1.8 times more likely to TB treatment loss to follow-up. In Brazil, the greatest social inequality is driven by income disparity with a wide heterogeneous distribution in the population, a fact that significantly impacts adherence to treatment and mortality from TB [32]. Thus, although a municipality may have a high HDI, it may also show great inequalities among its populations, a phenomenon that indicates risks for not completing TB treatment. Thus, policy strategies to cover health services that are in line with the social and economic development of cities may reduce known risk factors for TB treatment loss to follow-up.

TB surveillance based on territory [33] and literature that approach the relations of the individual and family with the social environment, such as the genogram and ecomap [34], stimulate pathways of success in the therapeutic process. We emphasise that at the national level, potential implementation of this clinical practice with the use of risk nomograms is an innovative and stimulating proposal for the development of the present study. Other researchers have also developed similar models to predict better TB treatment outcomes [21, 23, 35].

The study has some limitations. Although the utilised data were collected by the national surveillance service of transmissible diseases in Brazil, many variables with missing values were found. However, the data analysis was not compromised by this issue due to the importance of the theme worldwide and the magnitude of the sample under analysis. These factors allow the results to be amplified.

## 5 Conclusion

This study identified individual and ecological risk factors for abandoning TB treatment in patients in Sergipe, Brazil. The data revealed that individuals who are male, have brown or black skin, aged 20–39 years, have little schooling, live with AIDS, use alcohol and drugs, live in urban areas, are homeless or live in municipalities with high a HDI and high-income inequality are more likely to not adhere to TB treatment. The nomogram provides health professionals with an early identification of individuals who are at risk for treatment loss to follow-up.

Individuals with more risk factors should be viewed as more vulnerable populations; they would require differentiated treatment completion of treatment strategies. The health service and social conditions must guide the synergy of this movement to achieve different and better results. Therefore, it is necessary to monitor the individuals in a specific way with a balanced orientation and without risks to the completion of treatment process. We reinforce that the greatest capacity in the diagnosis and treatment service must be located where the need of the individual, family and community is greatest.

The social inequities, behavioral and epidemiological characteristics and existing comorbidities influence loss to follow-up. The completion of treatment for TB requires specific monitoring by health services and the identification of patients at increased risk for loss to follow-up is essential to improve health care and control of TB.

## Declarations

### Author contribution statement

Shirley Verônica Melo Almeida Lima and Carla Nunes: Conceived and designed the experiments; Analyzed and interpreted the data; Wrote the paper.

Karina Conceição Gomes Machado de Araújo and Marco Antonio Prado Nunes: Performed the experiments; Analyzed and interpreted the data; Contributed reagents, materials, analysis tools or data.

### Funding statement

This work was supported by 10.13039/501100002322Coordenação de Aperfeiçoamento de Pessoal de Nível Superior, Brazil, ID: 88881.187327/2018–01.

### Data availability statement

Data will be made available on request.

### Declaration of interests statement

The authors declare no conflict of interest.

### Additional information

No additional information is available for this paper.

## References

[1] WHO (2018). Global Report of Tuberculosis 2018.

[2] Ruffino-netto A. (2002). Tuberculose: a calamidade negligenciada/Tuberculosis: the negleted calamity. Rev. Soc. Bras. Med. Trop..

[3] (2019). Brasil. Guia de Vigilância em Saúde. *Ministério da Saúde, Secr Vigilância em Saúde, Dep Vigilância das Doenças Transm*.

[4] Lana F.C.F., Rodrigues F.G., Diniz M.B. (2003). Adesão ao tratamento/profilaxia de tuberculose associada à infecção HIV/AIDS no Centro de Treinamento e Referência em Doenças Infecciosas e Parasitárias. REME rev min enferm.

[5] Silva P da F., Moura G.S., Caldas A de J.M. (2014). Fatores associados ao abandono do tratamento da tuberculose pulmonar no Maranhão , Brasil , no período de 2001 a 2010. Cad. Saúde Pública.

[6] Costa-Veiga A., Briz T., Nunes C. (2018). Unsuccessful treatment in pulmonary tuberculosis: factors and a consequent predictive model. Eur. J. Publ. Health.

[7] Vicentin G., Santo A.H.C.M. (2002). Mortalidade por tuberculose e indicadores sociais no município do Rio de Janeiro. Ciência Saúde Coletiva.

[8] Ivaneide R., Larissa M., Régia P., Sílvio S. (2010). Abandono do tratamento de tuberculose em co-infectados TB/HIV. Rev. Esc. Enferm. USP.

[9] Brasil (2019). Brasil Livre da Tuberculose: evolução dos cenários epidemiológicos e operacionais da doença. Bol. Epidemiol..

[10] Brasil (2018). Panorama da tuberculose no Brasil. Diagnóstico situacional a partir de indicadores epidemiológicos e operacionais.

[11] Reid M.J.A., Arinaminpathy N., Bloom A. (2019). Building a tuberculosis-free world: the Lancet Commission on tuberculosis. Lancet (London, England).

[12] WHO (2018). Housing and Health Guidelines.

[13] (2019). IBGE. Brasil. *Inst Bras Geogr e Estatística - IBGE Cid Sergipe*.

[14] França V.L.A., Cruz M.T.S., Grafset Editora (2007). Atlas Escolar Sergipe - Espaço Geo Histórico e Cultural.

[15] UNDP (2018). Human development indices and indicators, statistical update. United Nations Dev Program.

[16] Gini C. (1912). Variabilita e Mutabilita : Contributo Allo Studio Delle Distribuzioni e Delle Relazioni Statistiche.

[17] Dahlgren G., Whitehead M. (2007). Policies and strategies to promote social equity in health background document to WHO.

[18] Solar O., Irwin A. (2010). A Conceptual Framework for Action on the Social Determinants of Health. Geneva.

[19] Santos R.V., Coimbra JCE a (2009). As causas sociais das iniquidades em saúde no Brasil.

[20] Marôco J. (2018). Análise Estatística Com O SPSS Statistics.

[21] Baussano I., Pivetta E., Vizzini L., Abbona F., Bugiani M. (2008). Predicting tuberculosis treatment outcome in a low-incidence area. Int. J. Tubercul. Lung Dis..

[22] Iasonos A., Schrag D., Raj G.V., Panageas K.S. (2008). How to build and interpret a nomogram for cancer prognosis. J. Clin. Oncol..

[23] Valdagni R., Rancati T., Fiorino C. (2008). Development of a set of nomograms to predict acute lower gastrointestinal toxicity for prostate cancer 3D-CRT. Int. J. Radiat. Oncol. Biol. Phys..

[24] (2017). Brasil. *Brasil Livre Da Tuberculose : Plano Nacional Pelo Fim Da Tuberculose Como Problema de Saúde Pública*.

[25] Lima S.V.M.A., Dantas A., Duque A.M. (2019). Spatial and temporal analysis of tuberculosis in an area of social inequality in Northeast Brazil. BMC Publ. Health.

[26] Luba T.R., Tang S., Liu Q., Gebremedhin S.A., Kisasi M.D., Feng Z. (2019). Knowledge, attitude and associated factors towards tuberculosis in Lesotho: a population based study. BMC Infect. Dis..

[27] Tola H.H., Karimi M., Yekaninejad M.S. (2017). Effects of sociodemographic characteristics and patients’ health beliefs on tuberculosis treatment adherence in Ethiopia: a structural equation modelling approach. Infect Dis Poverty.

[28] Gelmanova I.Y., Taran D.V., Mishustin S.P., Golubkov A.A., Solovyova A.V., Keshavjee S. (2011). ‘ Sputnik ’: a Programmatic Approach to Improve Tuberculosis Treatment Adherence and Outcome Among Defaulters SUMMARY.

[29] Barreto M.L. (2017). Desigualdades em Saúde: uma perspectiva global. Ciência Saúde Coletiva.

[30] Katz M.H. (2017). Homelessness—challenges and progress. JAMA, J. Am. Med. Assoc..

[31] Moreira T.R., Lemos A.C., Colodette R.M., Patrícia A., Siqueira R. (2019). Prevalência de tuberculose na população privada de liberdade: revisão sistemática e metanálise. Rev. Panam. Salud Públic.

[32] Alves J.D., Arroyo L.H., Moraes Arcoverde M.A. (2019). Magnitude of Social Determinants in the Risk of Death from Tuberculosis in Central-west Brazil.

[33] Leonor E., Maciel N., Maia C., Sales M. (2016). A vigilância epidemiológica da tuberculose no Brasil: como é possível avançar mais?. Epidemiol e Serviços Saúde..

[34] Nascimento L.C., Dantas IR de O., Andrade R.D., de Mello D.F. (2014). Genograma e ecomapa: contribuições da enfermagem Brasileira. Texto e Context Enferm.

[35] Rodrigo T., Caylà J.A., Casals M. (2012). A predictive scoring instrument for tuberculosis lost to follow-up outcome. Respir. Res..

